# Measuring the latent reservoir for HIV-1: Quantification bias in near full-length genome sequencing methods

**DOI:** 10.1371/journal.ppat.1010845

**Published:** 2022-09-08

**Authors:** Jennifer A. White, Joshua T. Kufera, Niklas Bachmann, Weiwei Dai, Francesco R. Simonetti, Ciara Armstrong, Jun Lai, Subul Beg, Janet D. Siliciano, Robert F. Siliciano

**Affiliations:** 1 Department of Medicine, Johns Hopkins University School of Medicine, Baltimore, Maryland, United States of America; 2 Howard Hughes Medical Institute, Baltimore, Maryland, United States of America; Vaccine Research Center, UNITED STATES

## Abstract

Antiretroviral therapy (ART) effectively inhibits HIV-1 replication but is not curative due to the persistence of a latent viral reservoir in resting CD4^+^ T cells. This reservoir is a major barrier to cure. Sequencing studies have revealed that the population of proviruses persisting in ART-treated individuals is dominated by defective proviruses that cannot give rise to viral rebound due to fatal defects including large deletions and APOBEC3-mediated hypermutation. Near full genome sequencing (nFGS) of individual proviruses is used in reservoir assays to provide an estimate of the fraction of proviruses that are intact. nFGS methods rely on a long-distance outer PCR capturing most (~9 kb) of the genome, followed by nested inner PCRs. The outer PCR is carried out at limit dilution, and interpretation of the results is based on the assumption that all proviruses are quantitatively captured. Here, we evaluate nFGS methods using the intact proviral DNA assay (IPDA), a multiplex digital droplet PCR assay that quantitates intact and defective proviruses with single molecule sensitivity using only short, highly efficient amplicons. We analyzed proviral templates of known sequence to avoid the additional complication of sequence polymorphism. With the IPDA, we quantitated molecular yields at each step of nFGS methods. We demonstrate that nFGS methods are inefficient and miss ~70% of full-length proviruses due to amplification failure at the initial outer PCR step. In contrast, proviruses with large internal deletions encompassing 70% of the genome can be quantitatively amplified under the same conditions. Accurate measurement of the latent reservoir of HIV-1 is essential for evaluating the efficacy of cure strategies, and the bias against full length proviruses in nFGS methods must be considered.

## Author summary

Despite antiretroviral therapy, HIV-1 persists in a small population of resting memory CD4^+^ T cells carrying a latent viral genome. This latent reservoir is the major barrier to cure. Accurate reservoir assays are critical for evaluating therapies aimed at reducing the reservoir. Sequencing studies have shown that defective proviruses greatly outnumber the intact, replication-competent proviruses responsible for viral rebound, and reservoir assays that rely on near full-genome sequencing (nFGS) have provided important qualitative information about intact and defective proviruses. However, it is assumed that all proviruses are amplified with equal efficiency in nFGS methods, regardless of sequence length. Here, we rigorously measure the efficiency with which nFGS methods detect intact and defective proviruses using a highly efficient multiplex digital droplet PCR assay, the intact proviral DNA assay. This assay allows direct counting of input proviral template molecules as well as PCR amplified products generated with different nFGS methods. We determined that nFGS methods do not provide an accurate quantitative measure of intact proviruses. Only ~30% of intact proviruses were detected, while proviruses with large internal deletions were amplified at expected frequencies. Our study demonstrates that nFGS methods do not provide accurate quantitative information about the size and composition of the latent reservoir.

## Introduction

The latent reservoir for HIV-1 consists of resting CD4^+^ T cells harboring replication-competent proviruses that are transcriptionally inactive while the cells remain in a resting state [1,2]. This reservoir persists despite treatment with antiretroviral therapy (ART) and precludes cure [3–6]. Accurate measurement of the reservoir is critical for evaluating interventions aimed at producing a cure such as the “shock and kill” strategy to eliminate latently infected cells [7–9], therapeutic vaccination [8] and broadly neutralizing antibodies [10,11] to prevent viral rebound, and combinations of such interventions [8]. Multiple reservoir assays exist [reviewed in Massanella and Richman, 2016 [12] and Abdel-Mohsen et al, 2020 [13]]. However, there are discrepancies between existing assays [14]. The reservoir was first defined with a quantitative viral outgrowth assay (QVOA) which directly measures the frequency of latently infected cells that are induced to produce infectious virus after a single round of *in vitro* T cell activation [2,3]. While the QVOA provides a definitive minimal estimate of the frequency of latently infected cells, it underestimates the true size of the reservoir because not all replication-competent proviruses are induced following a single round of T cell activation [15–17]. Even after multiple *in vitro* stimulations, less than 10% of cells carrying intact proviruses give rise to viral outgrowth [18].

While the QVOA underestimates reservoir size, PCR-based assays that amplify a single subgenomic region of the provirus such as *gag* greatly overestimate reservoir size because they fail to distinguish intact and defective proviruses. Analysis of the proviral landscape with near full genome sequencing (nFGS) of individual proviruses has revealed that the vast majority of proviruses persisting in treated individuals are defective [15,19–25]. The defects include large internal deletions, defects in the major splice donor (MSD) and packaging signal (ψ) sites, and APOBEC3G-mediated G→A hypermutation. In published nFGS studies, intact proviruses are reported to make up a minority (<10%) of all proviruses persisting in treated individuals [15,19–26]. Similar results have been reported for SIV, SHIV, and HIV-2 [27]. Most of the observed deletions are large, encompassing an average of almost 50% of the HIV-1 genome [20,27]. APOBEC3G-mediated hypermutation introduces multiple premature stop codons in most open reading frames [20,26,27]. Proviruses with these types of defects are unable to generate infectious virus and clearly should be excluded from reservoir measurements. Because defective proviruses greatly outnumber intact, replication-competent proviruses, assays that can distinguish and separately quantify intact proviruses are critical for evaluating HIV-1 eradication strategies. To this end, some reservoir assays measure the induction of viral protein expression [28], although some defective proviruses can also give rise to viral proteins [28,29]. An advantage of nFGS based methods is that they allow visualization of all defects evident at the primary sequence level.

One potential caveat with nFGS methods used to define the proviral landscape is that they depend on an initial long-distance PCR (~9 kb) carried out at limit dilution. In most nFGS studies, the efficiency of this PCR is not clearly defined. For nFGS assays to preserve accurate quantitation, they must capture the entire population of proviruses within a sample without bias [30]. Thus, it is paramount to consider PCR efficiency. Many factors influence the success of PCR including template length and GC content, primer length and GC content, melting temperature, reagent concentrations, dinucleotide repeats, and polymerase fidelity and processivity [31]. However, the most critical factor is typically amplicon length. The initial 9 kb PCR used in nFGS methods has the potential to be extremely inefficient due to polymerase dissociation [32]. Since shorter sequences are amplified with greater efficiency, it cannot be assumed that HIV-1 proviruses of different lengths are amplified equally. Subsequent steps in nFGS methods use an aliquot from the outer PCR for nested inner PCRs. However, the nested PCRs used in some nFGS methods are also ~9 kb in length and could be similarly inefficient. In addition, it cannot be assumed that the nested reactions will compensate for amplification failure in the long-distance outer PCR.

Because many different methods for reservoir analysis and quantitation use an initial 9 kb outer PCR, we evaluated the efficiency with which these methods amplify full-length intact proviruses and defective proviruses carrying large internal deletions or G➔A hypermutation. The methods evaluated here include the original nFGS method [15,20,21], a 5′ LTR-to-3′ LTR single genome amplification and direct amplicon sequencing method [19], the Full-Length Individual Proviral Sequencing (FLIPS) method [22], a full length HIV sequencing (FLIP-seq) assay [25], and Q4PCR [23]. The efficiency of these methods was evaluated with the recently described intact proviral DNA assay (IPDA) which uses multiplexed droplet digital PCR (ddPCR) to analyze individual proviruses and distinguish intact from defective proviruses without the need for long-distance PCR [26,33]. The principle of ddPCR is that single copies of the template can be quantitatively amplified using short highly efficient PCRs occurring in nanoliter-sized droplets. This allows direct digital counting of input template molecules [34]. The IPDA uses duplex PCRs to interrogate two regions that are frequently deleted or hypermutated in defective proviruses, the ψ/MSD site and the Rev-response element in the *env* gene. Intact proviruses give amplification for both regions while most, but not all, defective proviruses fail to give amplification for both regions [26]. Because it can detect proviruses at the single molecule level using short, high efficiency PCRs, the IPDA was used to directly quantitate input template molecules as well as the PCR amplified products generated with different nFGS methods. We also determined whether the amount of product generated would be apparent with the detection methods used in the relevant assays (gel electrophoresis, qPCR, or next-generation sequencing). Lastly, we quantified how much each method underestimates the total number of intact proviruses. This study is the first to rigorously evaluate the efficiency of single molecule detection in nFGS methods. Although nFGS methods have provided important qualitative information about the population of proviruses that persist in people living with HIV (PLWH), our study demonstrates that these methods do not provide accurate quantitative information about the size and composition of the latent reservoir.

## Results

### Use of ddPCR to quantitate proviral amplification in nFGS methods

**Fig 1:**
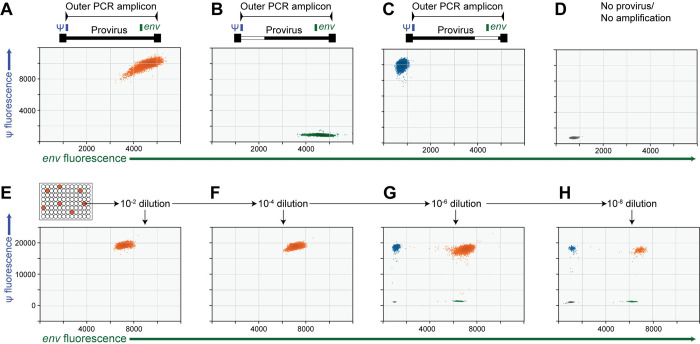
Use of the IPDA to evaluate PCR efficiency in nFGS methods. (**A-D**) IPDA analysis of PCR products from the outer PCR used in all nFGS methods (**S1 Table**). Template was DNA from a treated PLWH diluted to limit dilution with respect to proviruses. Each IPDA dot plot is shown below a diagram illustrating a possible proviral structure that would give rise to the observed plot. (**A**) Exponential amplification of an intact provirus. Each of the ~20,000 droplets in the reaction is positive for both the ψ and *env* amplicons. As a result, negative droplets which normally appear in the lower left quadrant are absent. (**B**) Exponential amplification of a provirus lacking the targeted *psi* sequence, for example due to a deletion such as that shown in the white box. (**C**) Representative well in which there was exponential amplification of a provirus lacking the targeted *env* sequence. (**D**) Representative negative well. (**E-H**) Use of the IPDA to quantitate molecular yields in nFGS methods. Positive PCRs from outer or nested reactions in nFGS methods are serially diluted until most droplets are negative. In this example, DNA from the J-Lat 6.3 clone was plated at limit dilution with respect to proviruses and amplified with the outer and nested PCRs of Method 4. An aliquot from a positive well was serially diluted and reanalyzed by IPDA. With more dilution, negative wells become dominant, allowing digital counting of PCR products as double positive droplets. Some shearing between IPDA amplicons is also evident in the form of single positive droplets at higher dilutions.

We made use of the single molecule sensitivity of the ddPCR [26,34] to assess the efficiency with which intact and defective HIV-1 proviruses are detected by nFGS methods. In preliminary experiments, we isolated DNA from CD4^+^ T cells of PLWH, measured the number of intact and defective proviruses using the IPDA, and then diluted the DNA to limit dilution with respect to total proviruses in 96 well plates. We then carried out the 9 kb outer PCR common to nFGS methods (see **S1 and S2 Tables**). The PCR products in each well were then analyzed by IPDA. The IPDA readily identified wells in which exponential amplification of a single intact provirus had occurred (**Fig 1A**). For these wells, each of the ~20,000 droplets analyzed was positive for both the ψ and *env* amplicons and likely contained multiple copies of the expected 9 kb PCR product. We also identified wells with exponential amplification of defective proviruses. In these wells, the dot plots showed only single positive droplets consistent with proviruses defective at the 5′ end (**Fig 1B**) or 3′ end (**Fig 1C**) of the genome. Because the proviruses were plated at limit dilution, the majority of the wells showed only a tight cluster of double-negative droplets (**Fig 1D**).

For reactions in which every droplet is positive, the yield of product molecules is likely much greater than the number of droplets, and quantitation of PCR products requires IPDA analysis of a very high dilution of the sample such that most droplets are negative. An example of this process is shown in **Fig 1E–1H**. At very high dilutions, most droplets are negative and the proviruses can be digitally counted as positive droplets. As is discussed in the next section, this analysis revealed enormous differences in the number of product molecules generated in different nFGS reactions, indicative of problems with PCR efficiency. Importantly, as is discussed below, the number of wells with amplification was below the value expected based on frequency of proviruses plated, also indicative of problems with PCR efficiency.

### Proviral sequence length variation in the relevant range directly impacts PCR efficiency and the number of product molecules generated

**Fig 2:**
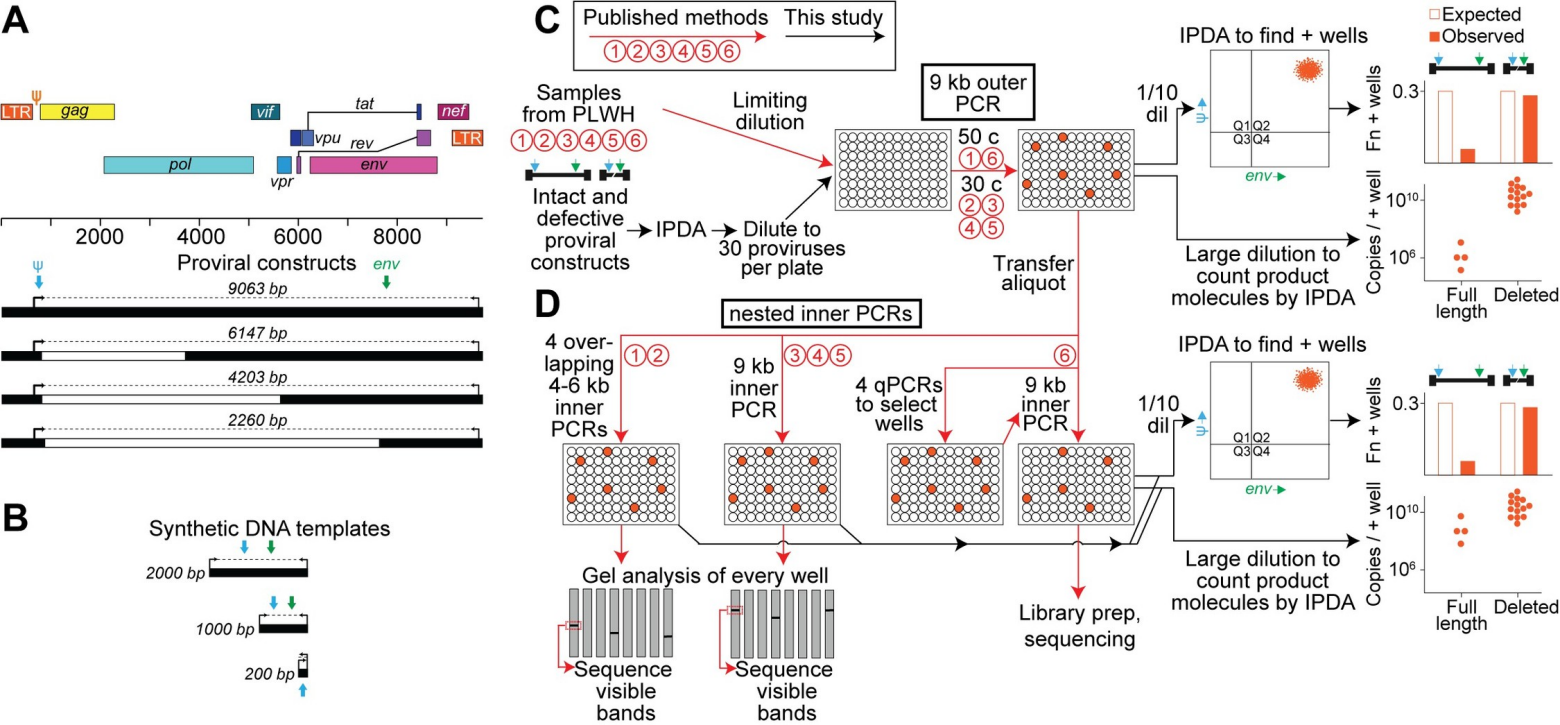
Quantitative analysis of nFGS methods. (**A**) Proviral constructs used to evaluate efficiency of nFGS methods. NL4-3-derived proviral constructs are shown in relation to the HIV-1 proviral map. Numbers in italics indicate the size of the amplicons obtained with the nFGS outer primers (black arrows). Deleted regions are shown in white. Positions of the IPDA ψ (blue arrows) and *env* (green arrows) amplicons are indicated. (**B**) Synthetic double stranded DNA templates used to evaluate efficiency of nFGS methods. (**C,D**) Experimental protocol. Published methods (red arrows) and our quantitative analysis of those methods (black arrows) are summarized in the flow diagram. Numbers in red circles refer to individual methods described in **S1 Table.** Analysis of the long-distance outer PCR step common to all nFGS methods is described in Panel **C.** Methods 1–6 all use the same outer PCR primers to generate a 9064 bp amplicon. Outer PCR wells were screened by IPDA analysis of a 1/10 dilution of the PCR products. The fraction of wells giving exponential amplification (see **Fig 1**) was determined, and then a large dilution of each positive well was analyzed by IPDA to count individual product molecules. The nested inner PCRs used in each nFGS method were analyzed as described in Panel **D**. For Method 6, a 9 kb inner PCR is run only for wells that have more than one positive nested subgenomic qPCR. Methods 3 and 5 are very similar to Method 4 but use a different polymerase and/or cycle number (see **S1 Table** for details) and were not tested here.

To quantify the efficiency with which nFGS methods detect HIV-1 proviruses, we used constructs representing an intact provirus and commonly observed forms of defective proviruses. We then diluted the constructs to limit dilution and evaluated the fraction of individual proviral templates that were successfully amplified by various nFGS methods and the number of product molecules generated in positive reactions (**Fig 2**). The HIV-1 reference provirus pNL4-3 [35] was used as the full-length intact provirus. In addition, we constructed plasmids containing the NL4-3 sequence with internal deletions of different sizes (**Fig 2A**). Constructs giving amplicon lengths of 6147 bp and 4203 bp were used to represent proviruses with deletions spanning 25% and 50% of the HIV-1 genome, respectively. Proviruses with very large internal deletions spanning 70% of the genome were represented by a construct giving a 2260 bp amplicon. These constructs span the range of deletion sizes observed in proviruses from treated individuals (average deletion size ~5 kb, reference 20). In addition, double stranded DNA gene fragments (gBlocks) were used as templates to generate amplicons of 2000 bp, 1000 bp, and 200 bp (**Fig 2B**). We also used the well characterized J-Lat cell line (clone 6.3), which carries a single integrated copy of an undeleted HXB2-derived provirus [36]. All of these proviral templates carry target sequences recognized by the IPDA primers and probes. The use of defined proviral templates of known sequence allowed us to evaluate nFGS methods under optimal conditions without the additional complication of sequence variability present in samples from PLWH.

Plasmids were linearized with a single restriction enzyme digest. Linearized plasmids, gBlocks, and DNA from J-Lat cells were then diluted into healthy donor DNA, and proviral template concentrations were checked by IPDA. Limiting dilutions are often established simply based on the frequency of positive wells following PCR amplification [30]. However, this approach fails to account for inefficiencies in the limiting dilution PCRs. Therefore, we used the IPDA to precisely measure the input template concentrations. Following IPDA quantitation, proviral templates were plated at a level of 30 proviruses per 96 well plate. Amplification was then carried out using the 9 kb outer PCR step common to all nFGS methods (**Fig 2C, S1 Table**).

**Fig 3:**
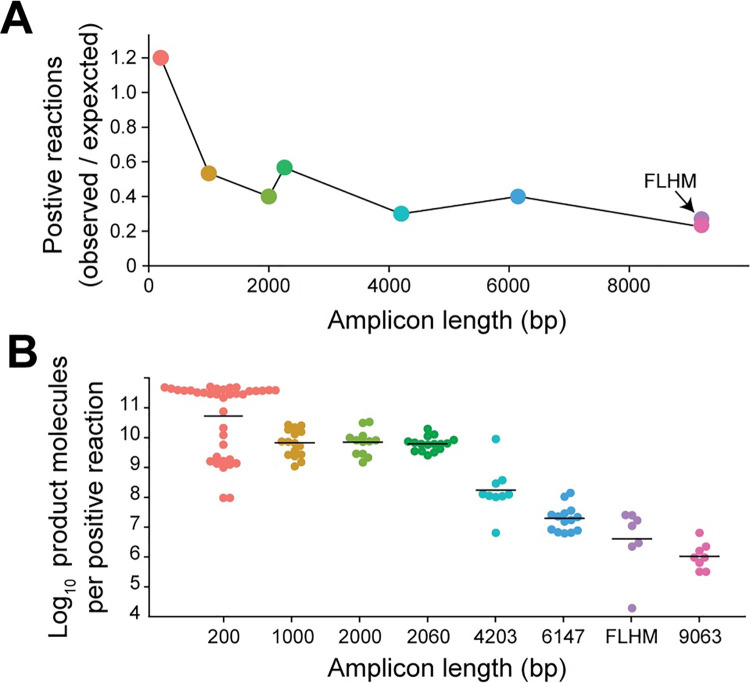
Proviral amplicon length affects both the fraction of limiting dilution PCRs with amplification and the yield of product molecules in positive reactions. (**A**) Effect of sequence length on the fraction of positive wells. Carefully quantitated NL4-3-derived proviral constructs of different lengths (**Fig 2A and 2B**) diluted into DNA from HIV-negative donors were plated at 30 proviruses per 96 well plate. A full length hypermutated (FLHM) provirus [p2g10, reference [29]] was also analyzed. After amplification by the outer PCR common to most nFGS methods, the fraction of positive wells was determined by IPDA analysis of each well. (**B**) Number of product molecules per reaction in the positive wells from **A** was determined by IPDA analysis of highly diluted aliquots from positive wells. Black lines show the geometric mean values.

Following amplification, screening by IPDA was carried out on a 1/10 dilution of each well (5 μl of each 50 μl reaction). Positive and negative wells were readily distinguishable as described above (**Fig 1**). The fraction of positive wells was close to the expected values for short amplicons (200 bp), but decreased dramatically for amplicons of 1 kb or larger **(Fig 3A**). For the full length proviral template, the fraction of positive wells was only 23% of the expected value. We also evaluated a full length hypermutated provirus (p2g10, reference 29) for which the fraction of positive wells was only 27% of the expected value (**Fig 3A**).

Using the IPDA, we also determined the number of product molecules generated in each positive outer PCR well. PCR products from positive wells were diluted such that the majority (>70%) of the droplets were negative to ensure that no more than one product molecule was partitioned into a single droplet. The number of product molecules generated in each positive outer PCR well was then calculated based on the number of double positive droplets detected in the IPDA and the fold dilution. In this manner, we demonstrated that proviral sequence length had a dramatic effect not only on the fraction of individual template molecules that were successfully amplified but also on the number of product molecules generated when amplification did occur **(Fig 3B**). Differences in amplicon length resulted in a striking 4–6 log difference in the number of product molecules produced from a single proviral template. The geometric mean number of molecules generated from successful amplification of a full-length intact provirus was 1.04 x 10^6^ (range, 3.20 x 10^5^ to 6.40 x 10^6^) while the geometric mean number of molecules generated from a short 200 bp sequence was 5.13 x 10^10^, with many values above 10^11^ (range, 9.60 x 10^7^ to 5.04 x 10^11^). This 4–6 log difference in molecular yield is much greater than the 50 fold mass difference and compromises detection of long products in mass based methods. Together, the results demonstrate that full length proviruses are amplified poorly in long distance PCRs, raising the possibility of a quantification bias in all nFGS methods.

### Long-distance PCR underestimates the frequency of full-length proviruses

**Fig 4:**
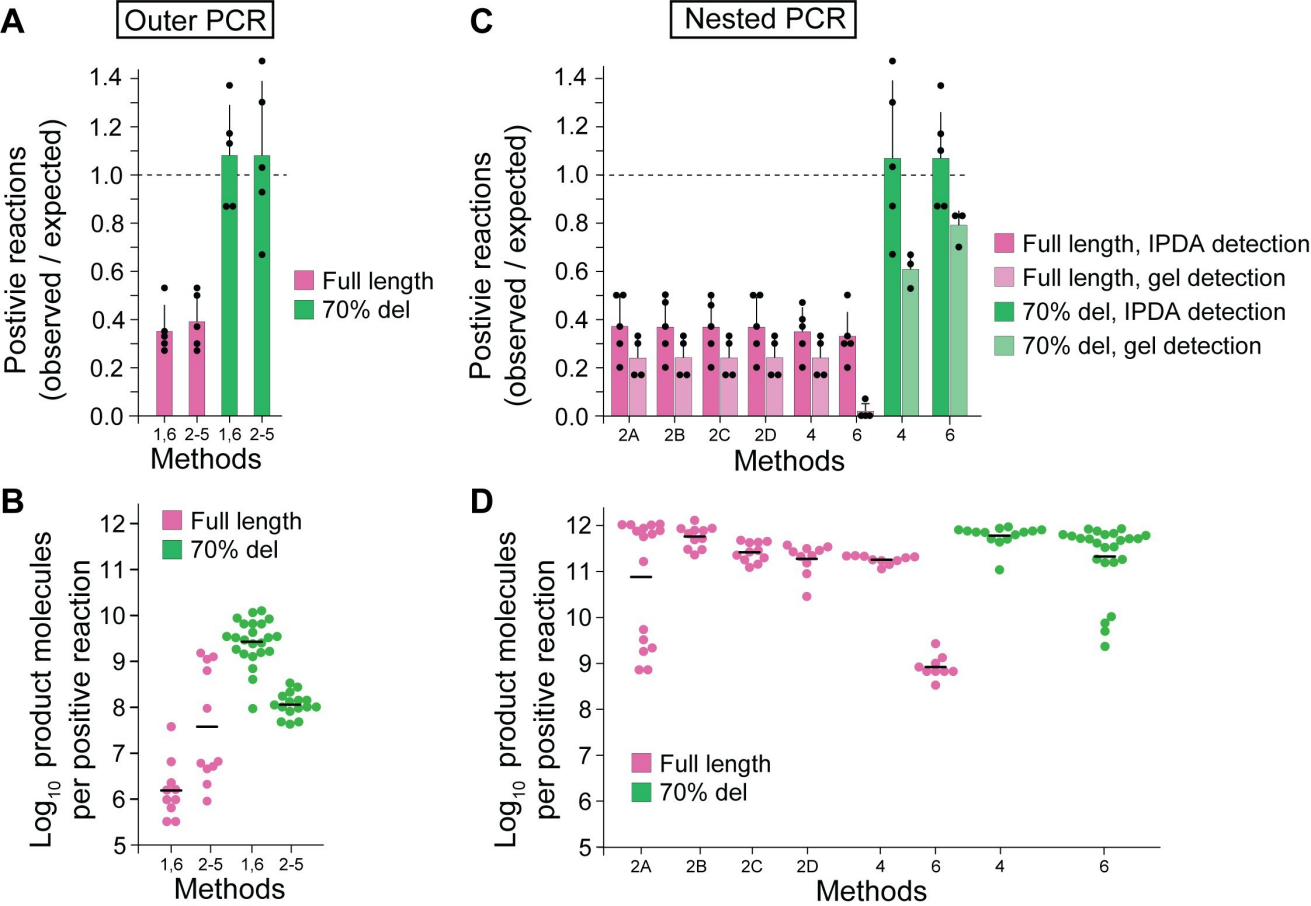
Amplification of full length and deleted proviruses by different nFGS methods. (**A**) Number of limiting dilution outer PCRs that are positive for amplification of full length and deleted proviruses by different nFGS methods. Carefully quantitated proviral constructs diluted into HIV-1 negative DNA were plated at 30 proviruses/96 well plate. After amplification under conditions used by Methods 1 and 6 or Methods 2–5 (see **S1 Table**), the fraction of wells with amplification was determined by IPDA. Results are expressed as the number of positive reactions per 96 well plate divided by the expected value (30/plate) for each of 5 plates (black circles). The means and standard deviations for the 5 plates tested per condition are shown as bars and black lines, respectively. The expected number of positive wells (dashed line) was observed for the proviral construct with a large internal deletion representing 70% of the genome (green bars) but not for the full-length provirus (pink bars). (**B**) Number of product molecules generated in the positive PCRs from **A**. Aliquots from positive wells from a representative plate were diluted extensively and analyzed by IPDA for digital counting of product molecules as described in **Fig 1E–1H**. Black lines show the geometric mean values. (**C**) Number of limiting dilution nested PCRs that are positive for full length and deleted proviral constructs amplified by different nFGS methods. After outer and nested amplification for under indicated conditions (see **Fig 2C and 2D** and **S1 Table**), the fraction of wells with amplification was determined by IPDA. Results are expressed as the fraction of positive wells relative to the expected value (30/plate) as described in **A**. In addition, 45 ul aliquots of nested PCR wells were analyzed by agarose gel electrophoresis, and the number of wells with visible bands was determined and plotted in the same manner (light colors). (**D**) Number of product molecules generated in the positive PCRs from a representative plate in **C**. Aliquots from positive wells were diluted extensively and analyzed by IPDA for digital counting of product molecules as described in **B**. Black lines show the geometric mean values.

To determine the extent by which nFGS methods underestimate the total number of intact proviruses, we compared amplification of a full length proviral construct (amplicon length = 9063 bp) and proviral construct with a large internal deletion (amplicon length = 2206 bp) by published nFGS methods (**S1 Table**) starting with a single template molecule per reaction. Each nFGS approach relies on an initial long-distance outer PCR using the same forward and reverse primers [15] with minor variations in amplification conditions (**S1 Table**). We diluted carefully quantitated proviral preparations to limit dilution as described above and amplified them using conditions specified in each of the published methods. We then determined the fraction of wells with exponential amplification after outer PCR and the number of product molecules generated in positive wells (**Fig 2C**). Positive and negative wells were readily distinguishable by IPDA as described in **Fig 1**. Based on the known number of copies plated in each outer PCR plate and the number of wells that were positive by IPDA following PCR amplification, we determined that nFGS methods that rely on long distance PCRs are extremely inefficient and fail to amplify the majority (61–65%) of full length (9 kb) proviruses (**Fig 4A, S3 Table**). For full length proviruses, the average fraction of positive wells from the outer PCR of Methods 1 and 6 was 35% ± 10% of the expected values based on five independent experiments. The average fraction of positive wells from the outer PCR of Methods 2–5 was 39% ± 11% of the expected values based on five independent experiments. In contrast, deleted proviruses giving a shorter amplicon length (2206 bp), representative of those with large internal deletions involving 70% of the genome, were amplified at ≥100% of the expected frequencies (**Fig 4A**). This result confirms the accuracy of the limiting dilutions and directly implicates amplicon length as a critical determinant of the ability of nFGS methods to detect individual proviruses.

Using the IPDA, we also determined the number of product molecules generated by the outer PCR of each nFGS method for reactions in which exponential amplification did occur. PCR products from positive wells were diluted such that the majority (>70%) of droplets were negative, ensuring that no droplet contained more than one proviral template. Amplification of intact genomes in the outer PCR of Methods 1 and 6 generated a geometric mean of 1.55 x 10^6^ molecules (range, 3.20 x 10^5^ to 3.74 x 10^7^) while the outer PCR of Methods 2–5 generated a geometric mean of 4.00 x 10^7^ molecules (range, 9.60 x 10^5^ to 1.60 x 10^9^) (**Fig 4B, S3 Table**). These values are 4–6 logs lower than the number of product molecules generated with short 200 bp amplicons (**Fig 3B**). Amplification of proviral constructs with a large 70% deletion using the outer PCR of the Methods 1 and 6 yielded a geometric mean of 2.76 x 10^9^ molecules (range, 9.60 x 10^7^ to 1.30 x 10^10^)(**Fig 4B**). This value was more than 3 logs higher than the number of molecules generated for intact proviruses. For Methods 2–5, the geometric mean was 1.12 x 10^8^ molecules (range, 4.20 x 10^7^ to 3.30 x 10^8^. Together, these results demonstrate that nFGS methods that rely on long-distance PCR amplify full-length proviral sequences poorly relative to proviruses with large deletions.

### Nested PCR

All nFGS methods use a nested inner PCR after the initial 9 kb outer PCR. To determine whether these nested reactions could compensate for the low efficiency observed with the outer PCRs, we subjected aliquots from all outer PCR wells to nested inner PCRs using the published protocols (**Fig 2D**). Methods 1 and 2 use four sets of nested inner PCRs (A-D) to obtain overlapping fragments that encompass most of the HIV-1 genome [15,20]. For the other methods, the nested PCR is a near full length (~9 kb) PCR capturing most of the HIV-1 genome (**S1 Table**). To determine the fraction of intact proviruses that are successfully amplified after the nested PCRs, aliquots of the nested PCR products were analyzed by IPDA (**Fig 2D**). For methods that use four overlapping nested PCRs, the average number of positive reactions was similar for each nested PCR, averaging 11 positive reactions per plate or 37% of the expected value (**Fig 4C**). This is similar to the fraction of wells that were positive after the outer PCR, reflecting the fact that the nested reactions are generally successful only if the initial outer PCR successfully amplifies the initial proviral template. Similar results were obtained for the methods that use a 9 kb nested inner PCR instead of four overlapping subgenomic PCRs. For example, amplification of a full length provirus with the outer and nested inner PCRs of Method 4, which is representative of Methods 3–5, yielded an average of 10.4 positive reactions/plate, 35% of the expected value and slightly below the fraction of positive wells in the outer PCR (**Fig 4A and 4C**). For Method 6, the 9 kb nested PCR yielded an average of 9.8 positive reactions/plate, or 32.7% of the expected value for intact proviruses. This is slightly below the values observed in the outer PCR (34.7% of expected). In contrast, when the 70% deletion construct was analyzed in the same manner with the outer and nested inner PCRs of Methods 4 and 6, the expected number of positive reactions was observed by IPDA (**Fig 4C**). Together these results demonstrate that even after nested inner PCRs, nFGS methods fail to amplify the majority of full-length proviruses under conditions that give quantitative detection of highly deleted proviruses. The failure to amplify intact proviruses is mainly due to amplification failure in the first outer PCR step.

The above experiments used the highly sensitive IPDA to detect amplification of the original template. However, in most published nFGS methods, successful amplification is detected by the presence of bands of appropriate size following gel electrophoresis. Therefore, we ran 45 μl of the 50 μl present in each nested PCR well on agarose gels. Nested PCRs from Methods 2A-D and Method 4 both generated an average of 7.3 visible bands per plate in four independent experiments (**Figs 4C and S1**). This is only 24.3% of the expected value, lower than the value detected by IPDA (**Fig 4C**). Therefore, the actual under-reporting of intact proviruses by published methods may approach 75%. Interestingly, while the 9 kb nested PCR from Method 6 generated enough product molecules to be detected by IPDA in an average of 9.8 wells per plate (32.7% of expected), only 2 wells in four total 96 well plates produced enough molecules to give visible bands upon agarose gel electrophoresis (1.6% of expected). Although Method 6 does not rely on detection of visible bands, these results raise concerns about the efficiency of detection of full-length proviruses by this method. Taken together, our results suggest that nFGS methods may fail to detect ~75% of full-length proviruses due to amplification failure in the first long distance outer PCR.

We corroborated the above results by directly quantitating the number of molecules produced by inner PCR amplification of intact proviruses with each nFGS method. This was done by IPDA analysis of highly diluted aliquots of PCR products from positive wells (**Fig 2C and 2D**). After the outer PCR, the nested PCRs of Method 2 generated geometric mean molecular yields 6.77 x 10^10^ to 5.07 x 10^11^ molecules/well (**Fig 4D**). The near full-length outer and nested inner PCRs from Method 4 generated a geometric mean of 1.8 x 10^11^ molecules/well (range, 1.14 x 10^11^ to 2.20 x 10^11^) (**Fig 4D**). In contrast, the near full-length outer and nested PCRs from Method 6 generated product yields that were more than 2 logs lower, with a geometric mean of 8.01 x 10^8^ molecules per positive reaction (range, 3.20 x 10^8^ to 2.56 x 10^9^). This result is consistent with the low detection of amplified products by gel electrophoresis (**Fig 4C**). For the proviral construct with a 70% deletion, Method 4 generated a geometric mean of 6.06 x 10^11^ molecules (range, 1.09 x 10^11^ to 9.43x 10^11^) while nested PCRs from Method 6 generated a geometric mean of 2.07 x 10^11^ (range, 2.28 x 10^9^ to 8.31 x 10^11^) (**Fig 4D**). Nested PCRs from Method 2 were not tested because the large internal deletion spanned the primer binding sites for both the forward and reverse primer sets. Overall, these results show that the nested PCRs generate reasonable yields if the initial 9 kb outer PCR is successful. However, the low rate of successful amplification in the initial 9 kb PCR generates a bias in the analysis of the proviral landscape by nFGS methods.

### Confirmation with J-Lat cells

**Fig 5:**
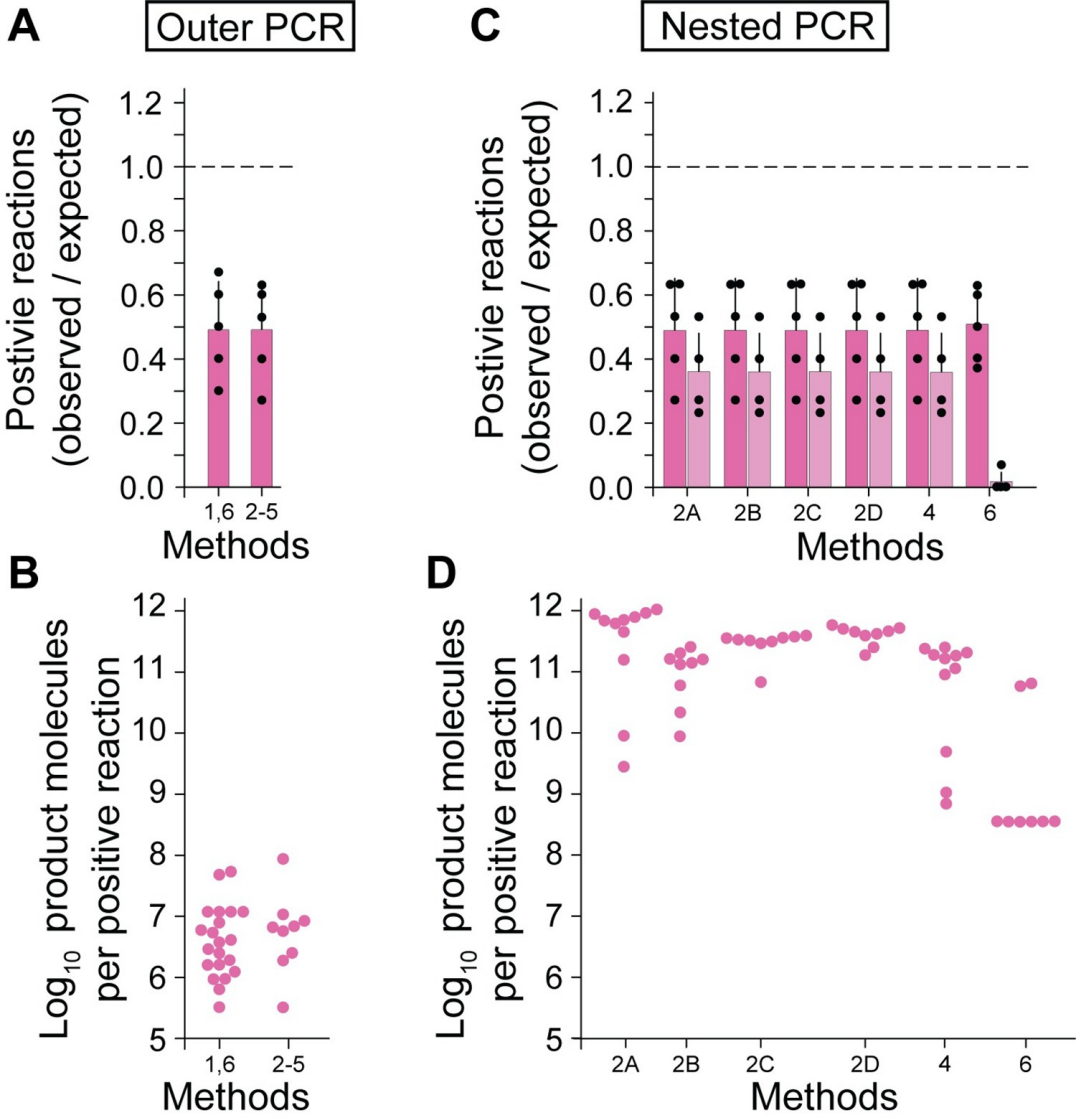
Amplification of intact proviruses from the J-Lat 6.3 cell line by different nFGS methods. (**A**) Number of limiting dilution outer PCRs that are positive for amplification of full-length proviruses by different nFGS methods. Carefully quantitated amounts of J-Lat DNA were diluted and plated at 30 intact proviruses/96 well plate. After amplification under conditions used by Methods 1 and 6 or Methods 2–5 (see **S1 Table**), the fraction of wells with amplification was determined by IPDA. Results are expressed as the number of positive reactions per 96 well plate divided by the expected value (30/plate) for each of 5 plates (black circles). The means and standard deviations for the 5 plates tested per condition are shown as bars and black lines, respectively. (**B**) Number of product molecules generated in the positive PCRs from **A**. Aliquots from positive wells from a representative plated were diluted extensively and analyzed by IPDA for digital counting of product molecules. Values from individual positive wells are shown as pink circles. (**C**) Number of limit dilution wells that were positive after nested PCR amplification by different nFGS methods. After outer and nested amplification under indicated conditions (see **Fig 2C and 2D** and **S1 Table**), the fraction of wells with amplification was determined by IPDA. Results are expressed as the fraction of positive wells relative to the expected value (30/plate) as described in **A** (dark pink bars). In addition, 45 ul aliquots of nested PCR wells were analyzed by agarose gel electrophoresis, and the number of wells with visible bands was determined and plotted in the same manner (light pink bars). (**D**) Number of product molecules generated in the positive PCRs from a representative plate in **C**. Aliquots from positive wells were diluted extensively and analyzed by IPDA for digital counting of product molecules as described in B. Values from individual positive wells are shown as pink circles.

To confirm the results obtained with intact proviral constructs, we used genomic DNA isolated from the J-Lat 6.3 cell line to determine the degree to which the frequency of intact proviruses is underestimated by nFGS methods. These cells contain a single full-length integrated provirus [36]. The number of proviruses present in J-Lat DNA samples was verified using the IPDA, and then the DNA was diluted into healthy donor DNA to limit dilution with respect to total proviruses before amplification (30 total proviruses per 96 well plate). The outer PCR from Method 1 and 6 gave an average of only 49% of the expected number of positive wells in five independent experiments (**Fig 5A**). Similar results were obtained with Methods 2–5. The number of molecules generated in each positive outer PCR was measured by IPDA (**Fig 5B**). The outer PCR of Methods 1 and 6 generated a geometric mean value of 2.71 x 10^6^ molecules per positive reaction (range, 3.20 x 10^5^ to 5.31 x 10^7^) while for Methods 2–5 the geometric mean value was 5.35 x 10^6^ molecules (range, 3.20 x 10^5^ to 8.86 x 10^7^). These values are very similar to those observed with full length proviral constructs but substantially lower than the values obtained for the construct with a large internal deletion (**Fig 4B**).

We also used the IPDA to measure the efficiency with which subsequent nested PCRs amplified templates generated in the outer PCR amplifications of J-Lat DNA. Overlapping nested PCRs (A-D) from Method 2 and the near full-length inner PCRs of Methods 4 and 6 all gave amplification for the same wells in which the outer PCR was positive (**Fig 5C**). As was the case with proviral constructs tested in **Fig 4**, amplification failure in the initial outer PCR limited detection of the J-Lat proviruses. Most methods except Method 6 use gel electrophoresis to detect positive wells after nested PCR. This method of detection is less sensitive than IPDA, and visible bands were observed at only 36% of the expected frequency based on five independent experiments (**Fig 5C**). Thus, based on analysis with J-Lat DNA, most nFGS methods miss over 60% of full-length proviruses.

Method 6 relies on a 9 kb nested inner PCR carried out on selected wells from the initial outer PCR (**Fig 2C and 2D**). This is followed by library preparation and next generation sequencing. When analyzed by gel electrophoresis, the number of visible bands generated by Method 6 was much lower than the number of bands produced by Methods 2 and 4. From four independent experiments, a total of only two bands were visible by gel using Method 6. This finding is consistent with IPDA measurements of the number of molecules generated in positive reactions (**Fig 5D**). The two wells that generated visible bands had higher molecular yields than the other six positive wells detected by IPDA. These results agree with the results obtained using NL4-3 as a template (only 2 bands in total visible by gel, **Fig 4C**) and raise concerns about the efficiency of the Method 6 for detecting full-length proviruses.

**Fig 6:**
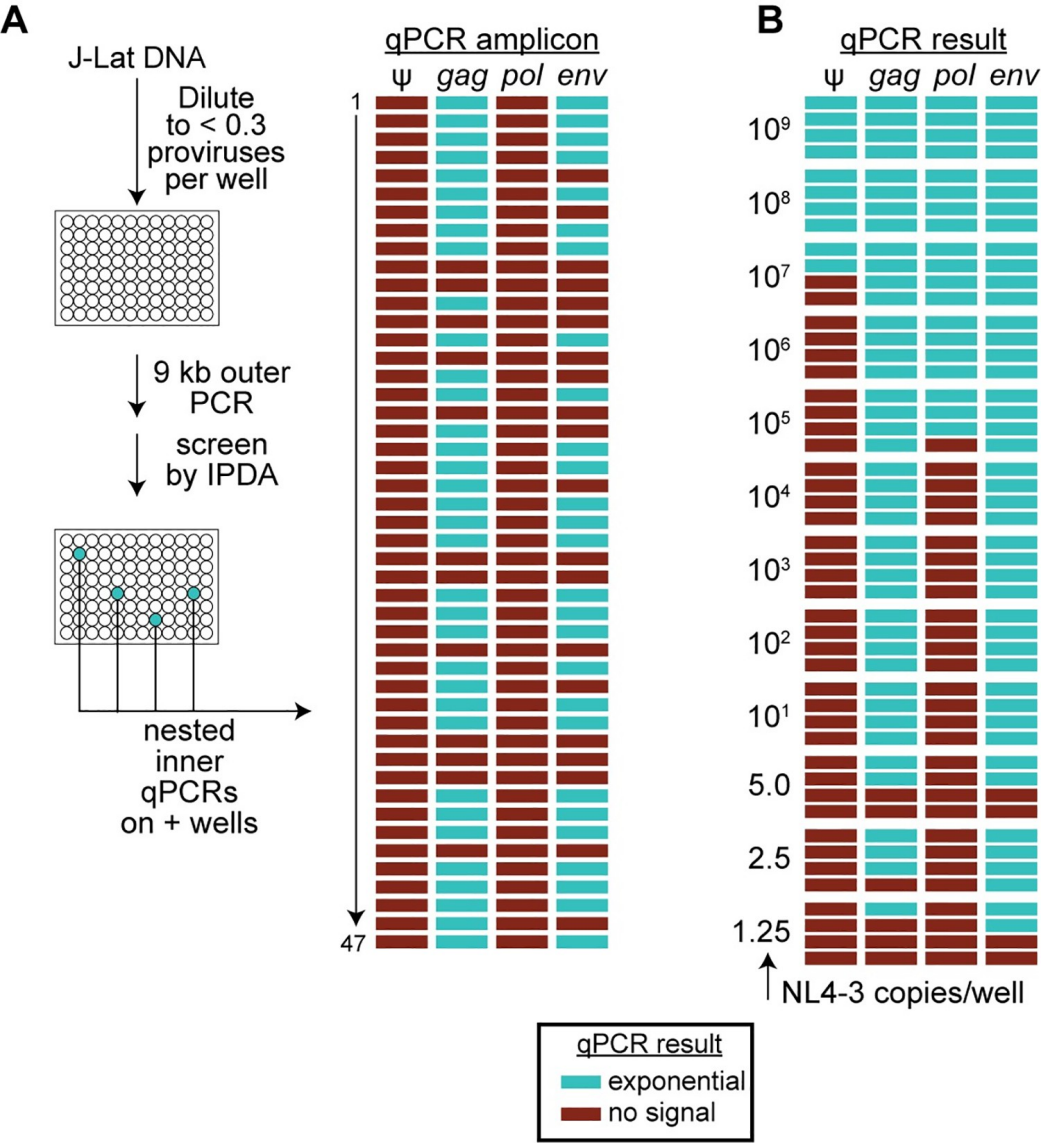
Efficiency of the nested qPCRs of Method 6. (**A**) Analysis with J-Lat cells. Carefully quantitated genomic DNA from J-Lat 6.3 cells was plated at limit dilution with respect to proviruses (30 proviruses/96 well plate). After the initial 9 kb outer PCR, wells were screened by IPDA. A total of 47 positive wells were identified in 3 independent experiments. Aliquots from these wells were subjected to 4 qPCR reactions used in Method 6. For each amplicon, successful qPCR amplification is indicated by a blue rectangle. (**B**) Analysis of qPCR efficiency with NL4-3. Carefully quantitated amounts of NL4-3 plasmid were diluted into HIV-negative donor DNA, plated at the indicated number of copies/well, and amplified using the four qPCRs of Method 6. For each amplicon, successful qPCR amplification is indicated by a blue rectangle.

An additional concern is related to the fact that Method 6 uses four nested inner qPCR reactions to screen outer PCR wells and determine which wells should be analyzed further (**Fig 2C and 2D**). Only wells that are positive for two or more of the four nested qPCR reactions are then subjected to the 9 kb nested inner PCR and sequenced [23]. Therefore, we also evaluated the efficiency of these qPCR reactions. Carefully quantitated genomic DNA from J-Lat 6.3 cells was plated at limit dilution with respect to proviruses (30 proviruses per 96 well plate). After the initial 9 kb outer PCR, each well was evaluated for amplification using the IPDA. In three independent experiments, a total of 47 positive reactions were identified by IDPA analysis. This value represents only 53% of the expected value for three plates (90 proviruses), consistent with the results presented above. However, the digital IPDA is more sensitive than the multiplexed qPCRs used to screen wells in Method 6. When the same 47 positive wells were analyzed with the four nested qPCR reactions of Method 6, only 27 of these wells (30% of expected) gave exponential amplification for two or more of the four qPCR amplicons (**Fig 6A**). Thus, by this analysis, Method 6 may miss 70% of intact proviruses. In three independent J-Lat experiments, none of the 47 wells tested were positive for the ψ amplicon despite the fact that all of these wells were positive by IPDA analysis, which uses the ψ amplicon in a digital droplet format. Thus, this qPCR reaction may be positive only if the number of templates generated in the outer PCR is high. The *pol* amplicon was also negative for these 47 wells. Eight of 47 wells were positive for only one probe and 12 wells had no signal from any of the four qPCR probes. The combined inefficiencies of the outer and nested qPCR reactions suggest that 70% of intact proviruses would escape detection by the Method 6.

The qPCRs used in Method 6 use as templates the products generated in the initial outer PCR. To understand how many molecules are required to achieve positive signal from all of the Method 6 qPCRs, we plated carefully quantitated amounts of NL4-3 plasmid diluted into HIV-negative donor DNA and carried out the qPCR reactions of Method 6 (**Fig 6B**). We tested NL4-3 concentrations ranging from 10^9^ copies per well to 1.25 copies per well. The median number of intact copies required to generate positive signals from all four qPCR reactions was 10^8^ copies per well. As the concentration of NL4-3 decreased, the first amplicon to fail was the ψ amplicon. Only two of four replicate wells with 10^7^ copies were positive. All reactions with fewer than 10^7^ copies were negative for the ψ amplicon. The median number of NL4-3 copies required for positive signals from three amplicons was 10^6^. The *pol* amplicon was the second to fail as template concentrations decreased. *Pol* signal was detected for three of the four replicates of 10^5^ copies per well but at concentrations below 10^5^ copies/well, no positive wells were observed. The *gag* and *env* amplicons performed better, with successful amplification observed with fewer than 10 copies/well. Taken together, these results show that the combined inefficiencies of long-distance PCR and qPCR further compromise accurate detection of intact proviruses in Method 6.

## Discussion

In this study, we used ddPCR to evaluate PCR efficiency of published nFGS methods. Previous studies used nFGS methods to show that the population of proviruses that persist in treated PLWH is dominated by proviruses with very large deletions and/or APOBEC3G-mediated hypermutation [15,19,20,22,24–26]. While these studies have provided extremely valuable qualitative insights into the proviral landscape, it is less clear that they should be used to provide quantitative measurements of the size and characteristics of the latent reservoir. These methods involve an initial near full-length (~9 kb) PCR carried out at limiting dilution, followed by nested inner PCRs and sequencing. A major caveat with the use of nFGS methods to measure the latent reservoir is that the efficiency of the initial 9 kb PCR has not been rigorously established. In contrast, the IPDA amplifies two short (116 bp), highly conserved regions of the HIV-1 genome and can directly quantify intact and defective proviruses at the single molecule level with high efficiency [26,33]. We used the IPDA to show here that reservoir assays that rely on long-distance PCR are biased against intact proviruses and fail to detect up to 75% of full-length proviruses due to amplification failure in the initial 9 kb outer PCR. In addition, we show that the nested PCRs used in all nFGS methods do not compensate for amplification failure in the initial outer PCR. If the outer PCR fails to give exponential amplification, the nested PCRs are negative. As an internal control, we also amplified a highly deleted construct giving a shorter 2 kb amplicon with the same methods and demonstrated quantitative detection of the deleted construct. Thus, it is paramount to consider PCR efficiency and the bias against full-length proviruses when nFGS methods are used to measure and characterize the latent reservoir for HIV-1.

To evaluate nFGS methods, we generated constructs representing an intact provirus and commonly observed forms of defective proviruses and used the IPDA to evaluate the fraction of each type of construct that was successfully amplified at limit dilution by various nFGS methods and the number of product molecules generated in positive reactions. We found that amplicon length had a dramatic effect not only on the fraction of individual template molecules that were successfully amplified but also on the number of product molecules generated when amplification did occur. For short amplicons (200 bp), the fraction of positive wells was close to the expected values, and the geometric mean number of molecules generated was 5.13 x 10^10^ (range, 9.60 x 10^7^ to 5.04 x 10^11^). In contrast, for a full-length intact provirus, the fraction of positive wells decreased dramatically, and the geometric mean number of molecules generated from successful amplification was 1.04 x 10^6^ (range, 3.20 x 10^5^ to 6.40 x 10^6^), 4–6 logs lower than for the short amplicons. These results demonstrate that full length proviruses are amplified poorly in long distance, limiting dilution PCRs, emphasizing a potential quantification bias in all nFGS methods.

Our analysis allowed us to determine that nFGS methods that rely on long distance PCR miss the majority of full-length proviruses due to amplification failure in the long first outer PCR step. For intact proviruses, outer PCRs of nFGS methods fail to amplify 61–65% of full-length (9 kb) proviruses. In contrast, proviruses with shorter amplicon length (2206 bp), representative of those with large internal deletions involving 70% of the genome, were amplified at the expected frequencies. We confirmed these results by using the IPDA to determine the number of product molecules generated in reactions where amplification did occur. With the outer PCR primers and conditions, amplification of a short 200 bp target sequence produced a molecular yield that was 4–5 logs higher than that generated with an intact provirus as template (**Fig 3B**). Amplification of a 2206 bp target sequence representing proviruses with a large 70% deletion using the outer PCR generated product molecule yields that were more than 2–3 logs higher than the number of molecules generated for intact proviruses (**Fig 4B**). Together, these results demonstrate that nFGS methods that rely on long-distance (9 kb) PCR are inefficient and amplify full-length sequences poorly relative to deleted proviruses.

It is important to note that the quantity and quality of the templates used in our study (NL4-3 plasmid and J-Lat DNA) were carefully assessed by IPDA prior to amplification by nFGS methods. This allowed us to determine the correct limit dilution and assess the fraction of individual template molecules that could be successfully amplified by these methods. The use of defined templates also eliminated the potentially confounding effects of sequence variation in our study [33]. Although most nFGS primers are chosen in highly conserved regions of the genome, it is possible that sequence variation in these regions could compound the problem of low PCR efficiency and further compromise the accuracy of reservoir measurements made using nFGS methods. It is also important to note that our analysis likely underestimates the actual quantification bias in nFGS methods because DNA shearing during isolation could introduce additional bias against full length proviruses in nFGS approaches in reservoir quantitation [30]. Unlike nFGS methods, the IPDA controls for shearing by measuring shearing of the host gene RPP30 between two amplicons separated by the same distance as the Ψ and *env* amplicons of the IPDA [26]. Thus, the shearing of genomic DNA is characterized as part of the IPDA procedure and corrected for. In contrast, shearing is not explicitly considered or controlled for in nFGS methods. Different DNA isolation methods used in nFGS protocols yield unknown amounts of shearing. The original nFGS methods (Methods 1 and 2) used a column free extraction method [15,20] which generated larger fragments of DNA. Method 4 uses direct lysis of cells with non-ionic detergent and proteinase K; no additional DNA purification is performed [22]. For Method 6, DNA is isolated with either a column-free method or phenol extraction [23]. Because DNA shearing from these nFGS methods has not been extensively quantified, the extent to which these methods miss full length proviruses due simply to DNA shearing between the outer PCR primer binding sites is unknown. This further complicates interpretation of nFGS methods that attempt to quantitate the latent reservoir. Thus, in addition to the PCR inefficiency problem defined here, DNA shearing may further increase the bias in nFGS methods as shearing between outer PCR primers is more likely with full length vs. deleted proviruses.

Our studies show that even after nested inner PCRs, nFGS methods fail to amplify the majority of full-length proviruses under conditions that give quantitative detection of highly deleted proviruses. While Methods 1 and 2 use four sets of nested inner PCRs (A-D) to obtain overlapping fragments that together encompass most of the HIV-1 genome [15,20], the other methods use a near full length (~9 kb) nested PCR (**S1 Table**). For Methods 1 and 2, the average number of positive reactions was similar for each nested PCR, averaging 11 positive reactions per plate or 37% of the expected value (**Fig 4C**), which is similar to the fraction of wells that were positive after the outer PCR. This may reflect the fact that the nested reactions are meant to enrich what is amplified in the outer reaction and are therefore generally only successful if the initial outer PCR amplification is successful. Similar results were obtained for the methods that use a 9 kb nested inner PCR. The nested inner PCR of Method 4, which is widely used and representative of other methods, yielded an average of 10.4 positive reactions/plate, 35% of the expected value, which was slightly below the fraction of positive wells in the outer PCR (**Fig 4A and 4C**). This slight decrease may be in part due to the additional inefficiency of 9 kb nested reaction. In contrast, when the 70% deletion construct was analyzed in the same manner with the outer and nested inner PCRs of Method 4, the expected number of positive reactions was observed (**Fig 4C**).

For Method 6, the 9 kb nested PCR yielded an average of 9.8 positive reactions/plate, or 32.7% of the expected value for intact proviruses. This is slightly below the values observed in the outer PCR (34.7% of expected). Again, these results provide additional evidence for the inefficiency of the near full-length PCR. In contrast, outer and nested PCR amplification of a construct with a 70% deletion by this method gave the expected number of positive reactions (**Fig 4C**). Method 6 uses four multiplexed inner qPCR reactions to screen outer PCR wells and determine which wells to analyze further (**Fig 2C and2D**). Only wells positive for two or more of the four nested qPCR reactions are subjected to the 9 kb nested inner PCR and sequencing [23]. To evaluate the efficiency of these qPCR reactions, carefully quantitated genomic DNA from J-Lat 6.3 cells was plated at limit dilution. After the initial 9 kb outer PCR, each well was evaluated by the four qPCRs described in Method 6. Exponential amplification of two or more of the four qPCR amplicons was observed for only 30% of wells containing a provirus (**Fig 6A**). In our analysis, the ψ and *pol* qPCRs were especially problematic. The combined inefficiencies of the outer and nested qPCR reactions of Method 6 should be considered when making quantitative conclusions about intact proviruses.

Together these results demonstrate that even after nested inner PCRs, nFGS methods fail to amplify the majority of full-length proviruses under conditions that give quantitative detection of highly deleted proviruses. For methods that use gel electrophoresis to detect amplified proviruses (Methods 1–5), the underestimate of full-length proviruses is even worse.

The biological significance of the quantitation bias uncovered here lies mainly in its implications for the evaluation of HIV cure strategies and for understanding the landscape of proviruses persisting in PLWH. The nFGS studies evaluated here provide definitive information on the types of proviral defects are generated during viral replication *in vivo*. However, the relative frequencies of intact proviruses and various types of defective proviruses may actually be different from frequencies reported in the nFGS studies. The fraction of intact proviruses determined by nFGS may be inaccurate due to failure of as many as 70% of full proviruses to amplify in the initial long-distance PCR. We show in Fig 3A that PCR detection of proviruses falls off dramatically as amplicon length increases from 200 to 1000 bp and beyond. The result is that the proportion of intact proviruses and defective proviruses with small deletions will be underestimated. Proviruses with very large internal deletions amounting to 70% or more of the genome can be captured efficiently by nFGS, and their relative abundance will be overestimated as a result of the inefficient amplification of longer proviruses. Many studies using nFGS methods that rely on long distance PCRs have published quantitative conclusions that are likely affected by this bias against full-length proviruses. This is particularly evident in published studies in which measured intact proviral frequencies are extremely low. For example, a recent study quantifying intact proviruses by Method 4 found that two participants had no detectable intact sequences in any CD4^+^ T cell subset tested [37]. Additionally, only a single intact provirus was detected in six other participants in the same study. This finding is likely to be due in part to the inefficiencies described here as evidenced in the published Method 4 optimization data, in which detection failed in almost 20% of wells plated with 20 intact copies/well. Only 5% of wells plated with one and two intact copies/well gave successful amplification [22]. Another recent study potentially affected by nFGS bias used Method 5 to evaluate proviral reservoirs in elite controllers compared to PLWH on suppressive ART [38]. This study found that the median frequency of intact HIV DNA in ART-treated individuals was only ~2/10^6^ PBMCs. This finding does not align with previous quantitation by IPDA of intact proviruses in treated PLWH (median 54 intact proviruses/10^6^ CD4^+^ T cells, references 20 and 33). This difference may be due in part to the use of PBMC rather than purified CD4^+^ T cells. However, it may also reflect inefficient detection of intact proviruses by nFGS-based methods.

Similar issues were found in studies using Method 6. In one recent study, no intact proviruses were detected in any of the time points for two participants [39]. From the same study, four additional participants had single timepoints at which no intact proviruses were detected. Even more concerning is the fact that for two participants (P10 and P11), no intact proviruses were detected at the first time point (1–9 months after ART initiation). Although sampling issues or a very low frequency of intact provirus could explain these results, previous studies have shown that intact proviruses make up the majority of proviruses during early ART suppression [40], and large-scale studies with the IPDA rarely identify subjects for whom no intact proviruses are present [33]. Multiplexing of four qPCRs could reduce the sensitivity of individual primer-probe sets that have a dim fluorophore and lower efficiency.

In summary, the inefficient amplification of full-length proviruses could lead to a significant underestimation of the frequency of intact proviruses, especially when only a limited number of nFGS sequences are obtained and the number of intact proviruses observed is low or zero. In this situation, it is difficult to assess the effect of curative interventions in a statistically meaningful way, and such nFGS studies should not be used to claim a reduction in or absence of intact proviruses (which are almost certainly present in all PLWH).

While our study has focused on HIV-1, some of the same nFGS methods utilizing long-distance outer PCR have been applied to the analysis of SIV persistence [27]. As with Method 6, the method described by Long et al. uses qPCR screening to determine proviral intactness and which outer PCR wells should be subsequently amplified and sequenced [41]. While we did not measure the efficiency of this method, it is likely that intact proviruses are underestimated based on the inefficiency of the outer PCR.

Recently, whole genome amplification (WGA) with Phi 29 polymerase has been used to obtain sufficient DNA for both integration site analysis and full proviral sequencing from single cells [42,43]. However, to our knowledge, quantitative studies that rigorously measure the frequency with which these methods successfully detect intact HIV proviruses are still lacking. Not all regions of the infected cell’s genome may be amplified equally. The approach described here, involving rigorous quantitation of the starting template and molecular yields by IPDA, could be applied to WGA methods in order to assess how well these methods detect individual proviruses.

The IPDA amplicons were positioned to maximize the discrimination between intact proviruses and those with the most common types of defects: large deletions and APOBEC3-mediated hypermutation [26]. This was done using data from the original nFGS studies (Methods 1, 2, and 3; references 15, 19, and 20), and it is important to consider whether the biases uncovered here could affect the choice of IPDA amplicon positions. Most of the deletions detected in nFGS studies are large (average size ~ 5 kb). Proviruses with large deletions can be readily differentiated from intact proviruses by digital droplet PCR. This discrimination is relatively insensitive to the positioning of amplicons. For two well-spaced amplicons, most deleted proviruses fail to give amplification at one or both positions and can thus be distinguished from intact proviruses. The positioning of the current IPDA amplicons were strongly influenced by two factors. First, in clade B infections, there is a unique class of proviruses with very small deletions in the region of the packaging signal. These are identified using the 5′ IPDA primer. These proviruses are near full length and are likely to be undercounted by nFGS methods, and it is particularly important that the 5’ amplicon is positioned as it in the current IPDA. The second factor is the need to discriminate between intact and hypermutated proviruses. The 3′ IPDA amplicon was chosen to capture a position that is very frequently mutated in hypermutated proviruses. For these reasons, it is unlikely that the biases described here will affect optimal positioning of IPDA amplicons, at least for clade B infections.

There are multiple limitations to our study. First, we did not analyze other factors that influence PCR efficiency, such as sequence polymorphism or the use different DNA polymerases. All but one of the methods tested uses Platinum Taq High Fidelity (Invitrogen). We did not test KAPA HiFi hot start polymerase used in Method 3. Second, as mentioned above, we did not characterize shearing of different DNA isolation methods. DNA shearing is likely to further increase the extent which nFGS methods underestimate intact proviruses.

Overall, our results suggest that any nFGS method that relies on a long-distance outer PCR is likely to underestimate intact proviruses by up to 75%. These findings raise concerns about the practice of using biased nFGS results to report quantitative changes in the frequency of intact proviruses in HIV cure studies. As we have shown, full-length PCRs fail to amplify the majority of intact proviruses and generate significantly fewer product molecules. In comparison, proviruses with large internal deletions are quantitatively amplified under the same conditions. Inefficiency of qPCRs used to screen for intact proviruses amplified in the outer PCR may further underestimate the frequency of intact proviruses. Thus, nFGS methods do not provide accurate quantitative information about the size and composition of the latent reservoir, and careful consideration to this problem should be given when interpreting nFGS results.

## Materials and methods

### Ethics statement

The Johns Hopkins Institutional Review Board and the University of California San Francisco Committee on Human Research approved this study. All participants provided written consent before enrollment.

### Study participants

Except where indicated, participants were HIV-1-infected adults on suppressive ART with undetectable plasma HIV-1 RNA levels (<50 copies per ml) for more than 6 months.

### Resting CD4+ T cell isolation

Peripheral blood mononuclear cells (PBMCs) were isolated by density centrifugation using FicollPaque PLUS (GE Healthcare Life Sciences) following manufacturer’s instructions. Total CD4^+^ T cells were then enriched from PBMCs using negative immunomagnetic selection using the EasySep Human CD4^+^ T-Cell Enrichment Kit (StemCell Technologies). Resting CD4+ T cells (CD69–, CD25– and HLA-DR–) were isolated using a second negative selection step (CD25-Biotin; Anti-Biotin MicroBeads; CD69 MicroBead Kit II; Anti–HLA-DR MicroBeads, all from Miltenyi Biotec).

### DNA isolation and quantification

DNA was extracted from resting CD4^+^ T cells using a protocol that minimizes fragmentation of genomic DNA (gDNA) (Qiagen Gentra Puregene Cell Kit). The Qubit 3.0 Fluorometer and Qubit dsDNA BR Assay Kit (Thermo Fisher Scientific) were used to measure DNA concentrations.

### IPDA

IPDA was performed as previously described [26]. The plasmid pNL4-3 (carrying an intact HIV-1 provirus) was obtained through the NIH AIDS Reagent Program, Division of AIDS, NIAID, NIH: HIV-1 NL4-3 Infectious Molecular Clone (pNL4-3) from Dr. Malcolm Martin. Plasmids with internal deletions of various sizes (25%, 50%, and 70% of the full-length genome) were made from pNL4-3 using the In-Fusion Mutagenesis kit (Takara Bio, Inc.). The deletion site of each plasmid was confirmed by sequencing. The plasmids retain the ψ and *env* sequences detected by the IPDA (**Fig 2A and 2B**). Plasmids were linearized by a single restriction digest at the ZraI recognition site and serially diluted to end point concentrations. The IPDA was used to measure exact copy number of intact genomes/uL and shearing ratios of each plasmid dilution. The IPDA was also used to measure PCR amplification of HIV-1 DNA in both the outer and nested PCR reactions. PCR products were diluted between 10 to 10^8^-fold and measured by IPDA to calculate PCR efficiency and molecules generated in each well.

### nFGS

We analyzed nFGS methods using published protocols (see S1 Table for references) with the exception that input proviral template concentrations were carefully determined by IPDA.

## Supporting information

S1 Fig Gel electrophoresis of inner PCR products from nFGS methods. (**A-D**) Gels of PCR products resulting from outer and nested inner PCR amplification of the intact provirus NL4-3 using Method 2. After the initial 9 kb outer PCR, 4 aliquots were taken from each well and amplified with the 4 inner nested PCRs (A-D, see S1 Table). After this PCR, 45 μl aliquots from each well were run on agarose gels. Figure shows gels for each of the subgenomic inners PCRs for the top half of a representative 96 well plate (wells A1-D12). Bands in the expected range of 4–7 kb (see **S1 Table**) were observed for wells in which the outer PCR was successful. (**E-F**) Amplicons generated from intact proviral templates using the outer and 9 kb nested inner PCRs of Method 4 and Method 6, respectively. The expected 9 kb bands were observed in some wells with Method 4, but rarely for Method 6. (**G-H**) Amplicons generated from a proviral construct with a deletion encompassing 70% of the genome using Method 4 and Method 6, respectively. Bands of the expected 2 kb size are observed for both methods.
(DOCX)

S1 Table PCR conditions for Methods 1–6. (DOCX)

S2 Table PCR Primers. (DOCX)

S3 Table Analysis of PCR efficiency and molecular yields of nFGS methods. (DOCX)
