# Supplementary material for: Measuring the latent reservoir for HIV-1: Quantification bias in near full-length genome sequencing methods
Source: PLoS Pathog. 2022 Sep 8;18(9):e1010845. doi: 10.1371/journal.ppat.1010845 (PMC9488763; doi:10.1371/journal.ppat.1010845)
Supplement: S1 Fig — (A-D) Gels of PCR products resulting from outer and nested inner PCR amplification of the intact provirus NL4-3 using Method 2. After the initial 9 kb outer PCR, 4 aliquots were taken from each well and amplified with the 4 inner nested PCRs (A-D, see S1 Table). After this PCR, 45 μl aliquots from each well were run on agarose gels. Figure shows gels for each of the subgenomic inners PCRs for the top half of a representative 96 well plate (wells A1-D12). Bands in the expected range of 4–7 kb (see S1 Table) were observed for wells in which the outer PCR was successful. (E-F) Amplicons generated from intact proviral templates using the outer and 9 kb nested inner PCRs of Method 4 and Method 6, respectively. The expected 9 kb bands were observed in some wells with Method 4, but rarely for Method 6. (G-H) Amplicons generated from a proviral construct with a deletion encompassing 70% of the genome using Method 4 and Method 6, respectively. Bands of the expected 2 kb size are observed for both methods. (DOCX) [file ppat.1010845.s001.docx]

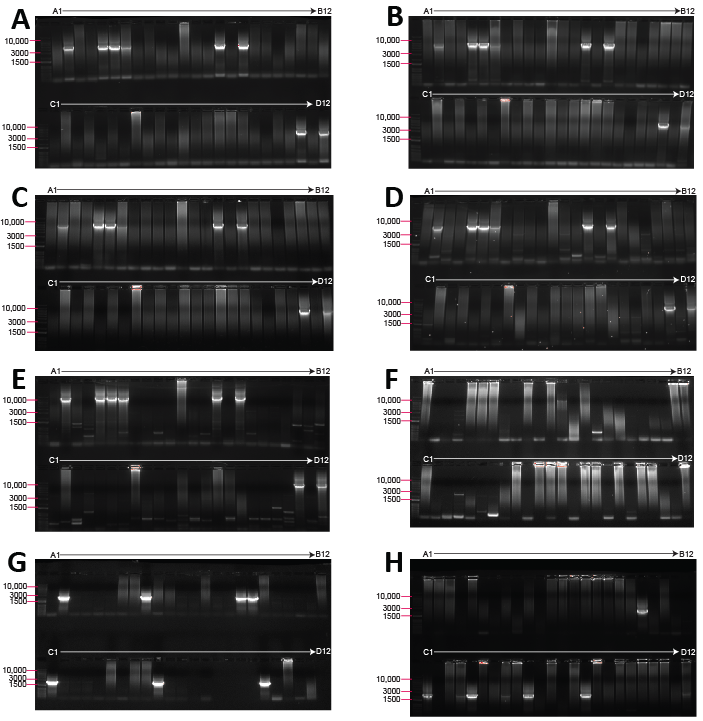


**S1 Fig.** Gel electrophoresis of inner PCR products from nFGS methods. (**A-D**) Gels of PCR products resulting from outer and nested inner PCR amplification of the intact provirus NL4-3 using Method 2. After the initial 9 kb outer PCR, 4 aliquots were taken from each well and amplified with the 4 inner nested PCRs (A-D, see Table S1). After this PCR, 45 μl aliquots from each well were run on agarose gels. Figure shows gels for each of the subgenomic inner PCRs for the top half of a representative 96 well plate (wells A1-D12). Bands in the expected range of 4-7 kb (see **S1Table**) were observed for wells in which the outer PCR was successful. (**E-F**) Amplicons generated from intact proviral templates using the outer and 9 kb nested inner PCRs of Method 4 and Method 6, respectively. The expected 9 kb bands were observed in some wells with Method 4, but rarely for Method 6. (**G-H**) Amplicons generated from a proviral construct with a deletion encompassing 70% of the genome using Method 4 and Method 6, respectively. Bands of the expected 2 kb size are observed for both methods.
