# Supplementary material for: Measuring the latent reservoir for HIV-1: Quantification bias in near full-length genome sequencing methods
Source: PLoS Pathog. 2022 Sep 8;18(9):e1010845. doi: 10.1371/journal.ppat.1010845 (PMC9488763; doi:10.1371/journal.ppat.1010845)
Supplement: S1 Table — (DOCX) [file ppat.1010845.s002.docx]

**S1 Table**. PCR conditions for Methods 1-6.

| Method^a^ | Name | Outer PCR^b^ | | | | Nested PCR^c^ | | | | Primer Conc. | Detection Method | Reference |
| --- | --- | --- | --- | --- | --- | --- | --- | --- | --- | --- | --- | --- |
|  |  | Cycles | HXB2 Position | Amplicon  Length (bp) | Extension  time | Cycles | HXB2 Position | Amplicon  Length (bp) | Extension  time |  |  |  |
| 1 | nFGS | 50 | 623-9686 | 9064 | 10 m | 45 | 646-5094 | 4449 | 5 m | 0.83 uM | Gel | Ho et al., 2013  (1) |
|  |  |  |  |  |  | 45 | 651-6443 | 5793 | 6 m | 0.83 uM |  |  |
|  |  |  |  |  |  | 45 | 3248-9632 | 6385 | 6 m 30 s | 0.83 uM |  |  |
|  |  |  |  |  |  | 45 | 4899-9676 | 4778 | 5 m | 0.83 uM |  |  |
| 2 | nFGS | 30 | 623-9686 | 9064 | 10 m | 45 | 646-5094 | 4449 | 5 m | 0.83 uM | Gel | Bruner et al., 2016  (2) |
|  |  |  |  |  |  | 45 | 651-6443 | 5793 | 6 m | 0.83 uM |  |  |
|  |  |  |  |  |  | 45 | 3248-9632 | 6385 | 6 m 30 s | 0.83 uM |  |  |
|  |  |  |  |  |  | 45 | 4899-9676 | 4778 | 5 m | 0.83 uM |  |  |
| 3^d^ | 5′ LTR-to-3′ LTR single genome amplification and direct amplicon sequencing* | 30 | 623-9686 | 9064 | 10 m | 30 | 682-9632 | 8951 | 10 m | 400 nM | Gel | Imamichi et al., 2016  (3) |
|  |  |  |  |  |  |  | 646-9676 | 9031 | 10 m |  |  |  |
| 4 | FLIPS | 30 | 623-9686 | 9064 | 10 m | 40 | 646-9676 | 9031 | 10 m | 1 uM | Gel | Heiner et al., 2017  (4) |
| 5 | Full Length HIV-1 Sequencing  (FLIP-seq) | 30 | 623-9686 | 9064 | 10 m | 30 | 638-9632 | 8995 | 10 m | 0.83 uM | Gel | Lee et al.,  2017  (5) |
| 6 | Q4PCR | 50 | 623-9686 | 9064 | 10 m | 50 | 646-9676 | 9031 | 10 m | 0.83 uM | Q4PCR | Gaebler et al., 2019  (6) |

^a^Genomic DNA isolation methods vary but for this study DNA was extracted using the QIAamp DNA Mini Kit (Qiagen) according to the manufacturer’s instructions. DNA concentrations were determined using the Qubit3.0 and Qubit dsDNA BR Assay Kit (ThermoFisher Scientific).

^b^Outer PCR cycling conditions: 94°C for 2 m; then 94°C for 30 s, 64°C for 30 s, 68°C for 10 m for 3 cycles; 94°C for 30 s, 61°C for 30 s, 68°C for 10 m for 3 cycles; 94°C for 30 s, 58°C for 30 s, 68°C for 10 m for 3 cycles; 94°C for 30 s, 55°C for 30 s, 68°C for 10 m for either 41 cycles for Method 1 and 6 (50 total cycles) or 21 cycles for Methods 2-5 (30 total cycles); then 68°C for 10 m.

^c^Inner PCR cycling conditions: (**X** = extension time for nested PCR from table above):94°C for 2 m; then 94°C for 30 s, 64°C for 30 s, 68°C for **X** m for 3 cycles; 94°C for 30 s, 61°C for 30 s, 68°C for **X** m for 3 cycles; 94°C for 30 s, 58°C for 30 s, 68°C for **X** m for 3 cycles; 94°C for 30 s, 55°C for 30 s, 68°C for **X** m for either 36 cycles for Method 1 and 2 (45 total cycles), 21 cycles for Method 3 and 5 (30 total cycles), 31 cycles for Method 4 (40 total cycles), or 41 cycles for Method 6 (50 total cycles); then 68°C for **X** m.

^d^This method used KAPA HiFi Hot Start polymerase.

References:

1. Ho Y-C, Shan L, Hosmane NN, Wang J, Laskey SB, Rosenbloom DIS, et al. Replication-Competent Noninduced Proviruses in the Latent Reservoir Increase Barrier to HIV-1 Cure. Cell 2013 Oct 24;155(3):540–51. https://doi.org/10.1016/j.cell.2013.09.020

2. Bruner KM, Murray AJ, Pollack RA, Soliman MG, Laskey SB, Capoferri AA, et al. Defective proviruses rapidly accumulate during acute HIV-1 infection. Nat Med 2016;22(9):1043–9. https://doi.org/10.1038/nm.4156

3. Imamichi H, Dewar RL, Adelsberger JW, Rehm CA, O’Doherty U, Paxinos EE, et al. Defective HIV-1 proviruses produce novel protein-coding RNA species in HIV-infected patients on combination antiretroviral therapy. Proc Natl Acad Sci 2016 Aug 2;113(31):8783-8788. http://www.pnas.org/content/113/31/8783.

4. Hiener B, Horsburgh BA, Eden J-S, Barton K, Schlub TE, Lee E, et al. Identification of Genetically Intact HIV-1 Proviruses in Specific CD4^+^ T Cells from Effectively Treated Participants. Cell Rep 2017 Oct 17;21(3):813–22. https://doi.org/10.1016/j.celrep.2017.09.081

5. Lee GQ, Orlova-Fink N, Einkauf K, Chowdhury FZ, Sun X, Harrington S, et al. Clonal expansion of genome-intact HIV-1 in functionally polarized Th1 CD4+ T cells. J Clin Invest 2017 Jun 30;127(7):2689–96. https://doi.org/10.1172/JCI93289

6. Gaebler C, Lorenzi JCC, Oliveira TY, Nogueira L, Ramos V, Lu C-L, et al. Combination of quadruplex qPCR and next-generation sequencing for qualitative and quantitative analysis of the HIV-1 latent reservoir. J Exp Med 2019 Jul 26;216(10):2253–64. https://doi.org/10.1084/jem.20190896
