# Supplementary material for: Measuring the latent reservoir for HIV-1: Quantification bias in near full-length genome sequencing methods
Source: PLoS Pathog. 2022 Sep 8;18(9):e1010845. doi: 10.1371/journal.ppat.1010845 (PMC9488763; doi:10.1371/journal.ppat.1010845)
Supplement: S2 Table — (DOCX) [file ppat.1010845.s003.docx]

**S2 Table**. PCR Primers^a^ (see S1 Table for references)

| Method | Outer  /inner | Amplicon | Name | HXB2 position | Sequence |
| --- | --- | --- | --- | --- | --- |
| 1 - 6 | Outer | nFL outer | BLOuterF | 623 – 649 | AAATCTCTAGCAGTGGCGCCCGAACAG |
|  |  | nFL outer | BLOuterR | 9,662 – 9,686 | TGAGGGATCTCTAGTTACCAGAGTC |
| 1,2 | Inner | A | 275F | 646 – 666 | ACAGGGACCTGAAAGCGAAAG |
|  |  |  | 3INOut | 5,072 – 5,094 | AATCCTCATCCTGTCTACTTGCC |
|  |  | B | 263F^b^ | 651 – 672 | GACTTGAAAGCGAAAGTAAAGC |
|  |  |  | 3AccOut | 6,421 – 6,443 | GGCATGTGTGGCCCARAYATTAT |
|  |  | C | 5INOut | 3,248 – 3,270 | ACTCCATCCTGATAAATGGACAG |
|  |  |  | BLInnerR | 9,604 – 9,632 | GCACTCAAGGCAAGCTTTATTGAGGCTTA |
|  |  | D | 5AccOut | 4,899 – 4,922 | CGGGTTTATTACAGGGACARCARA |
|  |  |  | 280R | 9,650 – 9,676 | CTAGTTACCAGAGTCACACAACAGACG |
| 3,4,6^c^ | Inner | nFL inner | 275F | 646 – 666 | ACAGGGACCTGAAAGCGAAAG |
|  |  |  | 280R | 9,650 – 9,676 | CTAGTTACCAGAGTCACACAACAGACG |

^a^Primers were desalted by the manufacturer.

^b^Custom primer to match NL4-3.

^c^Method 5 primers were not tested here.
