# Supplementary material for: Measuring the latent reservoir for HIV-1: Quantification bias in near full-length genome sequencing methods
Source: PLoS Pathog. 2022 Sep 8;18(9):e1010845. doi: 10.1371/journal.ppat.1010845 (PMC9488763; doi:10.1371/journal.ppat.1010845)
Supplement: S3 Table — (DOCX) [file ppat.1010845.s004.docx]

**S3 Table**. Analysis of PCR efficiency and molecular yields of nFGS methods­­­­­­.

| Method^a^ | Template |  | Outer PCR | | Inner PCR | | | | |
| --- | --- | --- | --- | --- | --- | --- | --- | --- | --- |
|  |  | Amplicon length (bp) | Fraction detected^b^  ± SD | Log_10_ copies per positive reaction  ± SD | Nested  amplicon | Amplicon length (bp) | Fraction detected by IPDA^b^  ± SD | Fraction detected by gel^b^  ± SD | Log_10_ copies per positive reaction  ± SD |
| 1 | NL4-3 | 9064 | 0.35 ± 0.11^c^ | 6.19 ± 0.66^c^ | ND^d^ | | | | |
| 2 | NL4-3 | 9064 | 0.39 ± 0.12^e^ | 7.60 ± 1.25^e^ | A^f^ | 4449 | 0.37 ± 0.13^g^ | 0.24 ± 0.08^g^ | 10.83 ± 1.33 |
|  |  |  |  |  | B | 5793 | 0.37 ± 0.12^g^ | 0.24 ± 0.08^g^ | 11.71 ± 0.25 |
|  |  |  |  |  | C | 6385 | 0.37 ± 0.12^g^ | 0.24 ± 0.08^g^ | 11.41 ± 0.21 |
|  |  |  |  |  | D | 4778 | 0.37 ± 0.13^g^ | 0.24 ± 0.08^g^ | 11.27 ± 0.34 |
| 4 | NL4-3 | 9064 | 0.39 ± 0.12^e^ | 7.60 ± 1.25^e^ | nFL^h^ | 9031 | 0.35 ± 0.10 | 0.24 ± 0.08 | 11.26 ± 0.09 |
| 6 | NL4-3 | 9064 | 0.35 ± 0.11^c^ | 6.19 ± 0.66^c^ | nFL | 9031 | 0.33 ± 0.11 | 0.02 ± 0.03 | 8.90 ± 0.27 |
| 1 | 70% del | 2206 | 1.08 ± 0.21 | 9.44 ± 0.49^c^ | ND^i^ | | | | |
| 2 | 70% del | 2206 | 1.08 ± 0.31 | 8.05 ± 0.26^e^ | ND^i^ | | | | |
| 4 | 70% del | 2206 | 1.08 ± 0.21 | 8.05 ± 0.26^e^ | nFL | 2173 | 1.07 ± 0.32 | 0.61 ± 0.06 | 11.78 ± 0.23 |
| 6 | 70% del | 2206 | 1.08 ± 0.31 | 9.44 ± 0.49^c^ | nFL | 2173 | 1.07 ± 0.21 | 0.79 ± 0.06 | 11.32 ± 0.76 |
| 1 | J-Lat | 9064 | 0.49 ± 0.15 | 6.43 ± 0.60^c^ | ND^d^ | | | | |
| 2 | J-Lat | 9064 | 0.49 ± 0.15 | 6.73 ± 0.66^e^ | A | 4449 | 0.49 ± 0.16^g^ | 0.36 ± 0.12^g^ | 11.37 ± 0.88 |
|  |  |  |  |  | B | 5793 | 0.49 ± 0.16^g^ | 0.36 ± 0.12^g^ | 10.90 ± 0.49 |
|  |  |  |  |  | C | 6385 | 0.49 ± 0.16^g^ | 0.36 ± 0.12^g^ | 11.42 ± 0.23 |
|  |  |  |  |  | D | 4778 | 0.49 ± 0.16^g^ | 0.36 ± 0.12^g^ | 11.56 ± 0.16 |
| 4 | J-Lat | 9064 | 0.49 ± 0.15 | 6.73 ± 0.66^e^ | nFL | 9031 | 0.49 ± 0.16 | 0.36 ± 0.12 | 10.63 ± 0.98 |
| 6 | J-Lat | 9064 | 0.49 ± 0.15 | 6.43 ± 0.60^c^ | nFL | 9031 | 0.51 ± 0.13 | 0.02 ± 0.03 | 9.06 ± 1.04 |

^a^See **S1 Table** for a description of each method. Conditions for the outer PCRs of Methods 1 and 6 are similar. Conditions for the outer PCRs of Methods 2-5 are similar.

^b^Positive reactions observed divided by positive reactions expected.

^c^Methods 1 and 6 use the same outer PCR conditions. Therefore, the same outer PCR reactions were used for analysis of both methods.

^d^ND, not done. The nested reactions for Method 1 are the same as the nested reactions for Method 2. Therefore, the Method 2 samples were used for this analysis.

^e^Methods 2 and 4 use the same outer PCR conditions. Therefore, the same outer PCR reactions were used for analysis of both methods.

^f^A-D are overlapping nested inner PCRs of Methods 1 and 2. See **S1 Table**.

^g^The fraction of nested inner PCR reactions that are positive is determined mainly by whether there is successful amplification in the outer PCR. For this reason, different inner PCRs using as template product from the same outer PCR give similar values for the fraction of positive reactions.

^h^Near full length nested inner PCR.

^i^Nested inner PCRs of Methods 1 and 2 were not done because the 70% deletion spanned binding sites for the nested inner primers.
